# Lipid droplets and polyunsaturated fatty acid trafficking: Balancing life and death

**DOI:** 10.3389/fcell.2023.1104725

**Published:** 2023-01-27

**Authors:** Mauro Danielli, Leja Perne, Eva Jarc Jovičić, Toni Petan

**Affiliations:** Department of Molecular and Biomedical Sciences, Jožef Stefan Institute, Ljubljana, Slovenia

**Keywords:** lipid droplet, fatty acid, ferroptosis, membrane remodeling, lipid oxidation, lipolysis, phospholipase

## Abstract

Lipid droplets are fat storage organelles ubiquitously distributed across the eukaryotic kingdom. They have a central role in regulating lipid metabolism and undergo a dynamic turnover of biogenesis and breakdown to meet cellular requirements for fatty acids, including polyunsaturated fatty acids. Polyunsaturated fatty acids esterified in membrane phospholipids define membrane fluidity and can be released by the activity of phospholipases A_2_ to act as ligands for nuclear receptors or to be metabolized into a wide spectrum of lipid signaling mediators. Polyunsaturated fatty acids in membrane phospholipids are also highly susceptible to lipid peroxidation, which if left uncontrolled leads to ferroptotic cell death. On the one hand, lipid droplets act as antioxidant organelles that control polyunsaturated fatty acid storage in triglycerides in order to reduce membrane lipid peroxidation, preserve organelle function and prevent cell death, including ferroptosis. On the other hand, lipid droplet breakdown fine-tunes the delivery of polyunsaturated fatty acids into metabolic and signaling pathways, but unrestricted lipid droplet breakdown may also lead to the release of lethal levels of polyunsaturated fatty acids. Precise regulation of lipid droplet turnover is thus essential for polyunsaturated fatty acid distribution and cellular homeostasis. In this review, we focus on emerging aspects of lipid droplet-mediated regulation of polyunsaturated fatty acid trafficking, including the management of membrane lipid peroxidation, ferroptosis and lipid mediator signaling.

## Introduction

Lipid droplets (LDs) are cytosolic organelles that are continuously synthesized and broken down in response to different cellular needs and environmental signals (Walther et al., 2016; Welte and Gould, 2017; Olzmann and Carvalho, 2019). Their unique and seemingly simple structure consisting of a neutral lipid core, composed mainly of triacylglycerols (TAGs) and sterol esters, covered by a single monolayer of phospholipids, has long concealed the dynamic nature of these organelles. LDs harbor numerous proteins and enzymes that not only participate in LD metabolism, but also enable LDs to interact with other organelles, metabolic processes and signaling pathways, thereby extending the function of LDs beyond lipids and energy production. However, even their most basic and well-recognized roles as regulators of lipid metabolism, fatty acid (FA) trafficking and signaling are still not fully understood (Walther et al., 2016; Henne et al., 2018; Olzmann and Carvalho, 2019; Jarc and Petan, 2020; Petan, 2020; Renne and Hariri, 2021).

Cells maintain a tight control of membrane composition by integrating pathways of *de novo* lipogenesis, lipid storage and removal to preserve membrane and organelle integrity and function. Accumulation of exogenous or endogenous free (unesterified) FAs in the cell leads to lipotoxicity, causing mitochondrial damage, endoplasmic reticulum (ER) stress, disruption of membrane integrity and cell death (Herms et al., 2013; Han and Kaufman, 2016; Petan et al., 2018; Olzmann and Carvalho, 2019). LDs have the unique ability to incorporate excess FAs and cholesterol into their neutral cores, packaged in the form of TAGs and cholesterol esters (CEs), thereby reducing potential lipotoxic damage (Greenberg and Coleman, 2011; Nguyen et al., 2017). LD accumulation is often triggered by nutrient and oxidative stress (Listenberger et al., 2003; Welte and Gould, 2017; Henne et al., 2018; Jarc et al., 2018; Jarc and Petan, 2019; Olzmann and Carvalho, 2019; Petan, 2020; Smolič et al., 2021; Wilcock et al., 2022), suggesting a tight coupling between energy and redox imbalances and LD turnover. The functions of LDs in stressed cells are influenced by the metabolic characteristics of the particular cell type and the current state of the cell, which is largely determined by nutrient and oxygen availability in the microenvironment. Notably, emerging evidence suggests that many of the roles of LDs in stressed cells involve managing membrane remodeling and composition, especially through the trafficking of saturated, monounsaturated and polyunsaturated FAs (PUFAs) among different lipid classes stored in various lipid pools and organelles.

Elevated lipid peroxidation of membrane-residing PUFAs, coupled with reduced cellular antioxidant capacity, leads to the accumulation of lipid peroxides and triggers ferroptosis, a type of regulated, iron-dependent cell death (Dixon et al., 2012). The relationship between LDs and the control of lipid peroxidation in ferroptosis is emerging (Bailey et al., 2015; Bai et al., 2018; Angeli et al., 2019; Petan, 2020; Aldrovandi et al., 2021; Lange and Olzmann, 2021; Subramanian and Marelli-Berg, 2021; Liang et al., 2022). LDs could be involved in ferroptosis through distinct and context dependent mechanisms, such as sequestering membrane-derived or exogenous PUFAs to prevent lipid peroxidation, or by providing PUFAs for phospholipid synthesis and thereby increasing membrane oxidative damage. LDs may also regulate membrane unsaturation by balancing the provision of monounsaturated fatty acids (MUFAs) for phospholipid synthesis and remodeling (Ackerman et al., 2018; Jarc et al., 2018). In addition, LDs are also storage pools for lipid signaling molecules. LDs can release PUFAs, which may directly bind to regulatory proteins and activate signaling pathways, or serve as precursors for the synthesis of bioactive lipid mediators, such as eicosanoids (Dvorak et al., 1983; Triggiani et al., 1994; Bozza et al., 2011; Dichlberger et al., 2014; Papackova and Cahova, 2015; Schlager et al., 2015; Schlager et al., 2017).

The idea that LDs play a major role in the subcellular distribution of PUFAs is rapidly evolving. In this review, we focus on the context-dependent, dual role of LDs in PUFA trafficking and metabolism, which can either protect against or contribute to membrane and organelle dysfunction and cell death.

## The Janus face of polyunsaturated fatty acids

PUFAs are essential FAs with more than one double bond. Linoleic (LA, 18:2ω-6) and α-linolenic (ALA, 18:3ω-3) acids are precursors for *de novo* synthesis of other long-chain PUFAs, including arachidonic acid (AA, 20:4ω-6), docosahexaenoic acid (DHA, 22:6ω-3) and eicosapentaenoic acid (EPA, 20:5ω-3). LA and ALA are not endogenously synthesized in mammals due to a lack of specific desaturases and need to be obtained from the diet (Ponnampalam et al., 2021). Several studies from recent years have shown that PUFA uptake and endogenous conversion are not redundant but rather support the diversity in PUFA species, which is required for proper function of several tissues (brain, metabolic tissues, reproductive organs, blood cells, etc.) and specific cellular mechanisms (osteogenesis, mechanosensing, etc.) (Moon et al., 2009; Roqueta-Rivera et al., 2010; Fan et al., 2012; Bazinet and Layé, 2014; Pauter et al., 2014; Stoffel et al., 2014; Levental et al., 2017; Hashimoto and Hossain, 2018). PUFA metabolism is regulated by numerous proteins and enzymes, including FA-binding proteins, elongases, desaturases, acyltransferases, acyl-CoA synthetases and esterases, some of which show pronounced preferences for PUFAs relative to other FAs. For example, phospholipase A_2_ (PLA_2_) and lysophospholipid acyltransferase (LPLAT) enzymes act together within the Lands’ cycle as membrane remodeling enzymes that allow phospholipids to go through a quality control circuit, whereby acyl chains are replaced by sequential PLA_2_/acyltransferase reactions. However, the mechanisms that control the selective incorporation of PUFAs into different cellular lipid pools, such as membrane phospholipids and TAGs, and the subcellular (re)distribution of PUFAs derived from various sources, e.g., from FA uptake or *de novo* synthesis (Young et al., 2022), are only beginning to emerge.

PUFAs confer a number of biophysical properties to cell membranes, including flexibility, fluidity, thickness and bending rigidity (Hyvönen and Kovanen, 2005; Antonny et al., 2015; Budin et al., 2018; Harayama and Shimizu, 2020; Tiberti et al., 2020). PUFAs are required for fundamental membrane functions, such as stabilization of membrane proteins, membrane fission during endocytosis, protein-lipid interactions and lipid peroxidation (Pinot et al., 2014; Vásquez et al., 2014; Stockwell et al., 2017; Lorent et al., 2020). These functions highlight the importance of a finely tuned control of PUFA content within cell membranes (Harayama and Shimizu, 2020). Membrane PUFAs also undergo enzymatic and non-enzymatic oxidation, which may lead to oxidative chain reactions that propagate within the membrane. Unrestricted membrane lipid peroxidation can result in organelle dysfunction and loss of plasma membrane integrity committing cells to ferroptosis (Dixon et al., 2012; Yang et al., 2014; Aldrovandi et al., 2021; Liang et al., 2022). The (patho)physiological roles of ferroptosis and the mechanisms that prevent this recently discovered form of cell death are currently under investigation. Elaborate surveillance and repair mechanisms that control PUFA peroxidation and prevent ferroptosis are emerging. The main one is mediated by the glutathione peroxidase 4 (GPX4) enzyme, which directly eliminates lipid peroxides formed in membrane phospholipids (Ursini et al., 1982; Liang et al., 2022). Other mechanisms that prevent ferroptosis include free radical scavengers generated by ferroptosis suppressor protein 1 (FSP1), dihydroorotate dehydrogenase (DHODH) and guanosine triphosphate cyclohydrolase 1 (GCH1) (Bersuker et al., 2019; Doll et al., 2019; Kraft et al., 2020; Soula et al., 2020; Mao et al., 2021). However, how lipid metabolism and PUFA trafficking are controlled to prevent or mitigate lipid peroxidation in response to ferroptotic insults is poorly understood.

PUFAs are also important signaling lipids and precursors for bioactive lipid mediators. They can directly affect the expression of genes by binding to and regulating the activity of different nuclear transcription factors, including peroxisome proliferator-activated receptors (PPARs), retinoid X receptors (RXRs) and sterol regulatory element-binding proteins (SREBPs), thereby playing a major role in FA, TAG and cholesterol metabolism (Xu et al., 1999; Lengqvist et al., 2004; Zúñiga et al., 2011; Papackova and Cahova, 2015; Echeverría et al., 2016; Harayama and Shimizu, 2020). PUFA oxidation with lipoxygenase (LOX) and cyclooxygenase (COX) enzymes leads to the production of lipid mediators, including prostaglandins and leukotrienes, which are secreted from cells in minute amounts to modulate various physiological functions, including cell growth, inflammation and immune responses (Wang and DuBois, 2010; Buckley et al., 2014; Serhan, 2014; Dennis and Norris, 2015; Chiurchiù et al., 2018). However, LOX enzymes have also been implicated in membrane phospholipid peroxidation leading to ferroptotic cell death (Yang et al., 2016; Kagan et al., 2017), suggesting that PUFA delivery into signaling vs. cell death oxygenation pathways must be tightly controlled. Lethal lipid peroxidation caused by LOX-mediated PUFA oxidation at the membrane occurs under specific conditions when major cellular redox mechanisms are dysregulated, e.g., by inhibition of GPX4 activity and/or glutathione deficiency (Yang et al., 2016; Kagan et al., 2017). On the other hand, conventional COX- and LOX-mediated lipid mediator synthesis is regulated by stimulus-induced PLA_2_-mediated membrane hydrolysis that controls the availability of free PUFAs (Lambeau and Gelb, 2008; Wang and DuBois, 2010; Murakami et al., 2022). In addition, these PUFA-derived lipid mediators may also be reincorporated into phospholipids to perform specific functions in the membrane (Hajeyah et al., 2020) and possibly serve as a readily available pool of lipid mediators. In addition, ferroptotic cells also produce and release bioactive lipid mediators (Angeli et al., 2014). Thus, PUFA oxygenation pathways have pleiotropic roles ranging from membrane homeostasis, metabolism, intra- and intercellular signaling, inflammation and immunity to the management of cell death.

The abundance of PUFA-containing phospholipids in the membrane has been recognized as an important factor that may tilt the redox balance against or in favor of lipid peroxidation (Rysman et al., 2010; Bailey et al., 2015; Doll et al., 2017; Agmon et al., 2018; Dierge et al., 2021; Bartolacci et al., 2022). Accordingly, several enzymes involved in membrane remodeling, including acyl-CoA synthetase long chain family member 4 (ACSL4), lysophosphatidylcholine acyltransferase 3 (LPCAT3), the group IV cytosolic PLA_2_ (cPLA_2_α) and the group VIA Ca^2+^-independent phospholipase A_2_ (iPLA_2_β) have already been identified as regulators of ferroptosis sensitivity (Doll et al., 2017; Kagan et al., 2017; Beharier et al., 2020; Sun et al., 2021; Bartolacci et al., 2022; Stockwell, 2022). However, we are far from an integrative view of how membrane remodeling is linked to other lipid metabolic pathways including *de novo* lipogenesis, lipid uptake or LD turnover (Petan, 2020; Bartolacci et al., 2022; Petan and Manček-Keber, 2022). Acting as dynamic buffering systems that control the delivery of FAs to various cellular destinations, LDs are emerging as key modulators of membrane composition and integrity, particularly in stressed cells. The channeling of PUFAs from membrane phospholipids and sequestration into LDs has been shown to protect cells from lipid peroxidation and oxidative stress (Bailey et al., 2015; Jarc et al., 2018). However, elevated PUFA release through LD breakdown can also induce oxidative stress and promote lipid peroxidation, suggesting that the precise regulation of LD turnover is essential for membrane and redox homeostasis (Wang et al., 2015; Bülow et al., 2018; Zheng et al., 2017; Bai et al., 2018; Jarc et al., 2018; Zou et al., 2019; Tousignant et al., 2020; You et al., 2021). The underlying mechanisms of LD-mediated control of membrane composition, lipid peroxidation and cell fate are yet to be uncovered.

## Lipid droplets as stress response organelles

**FIGURE 1:**
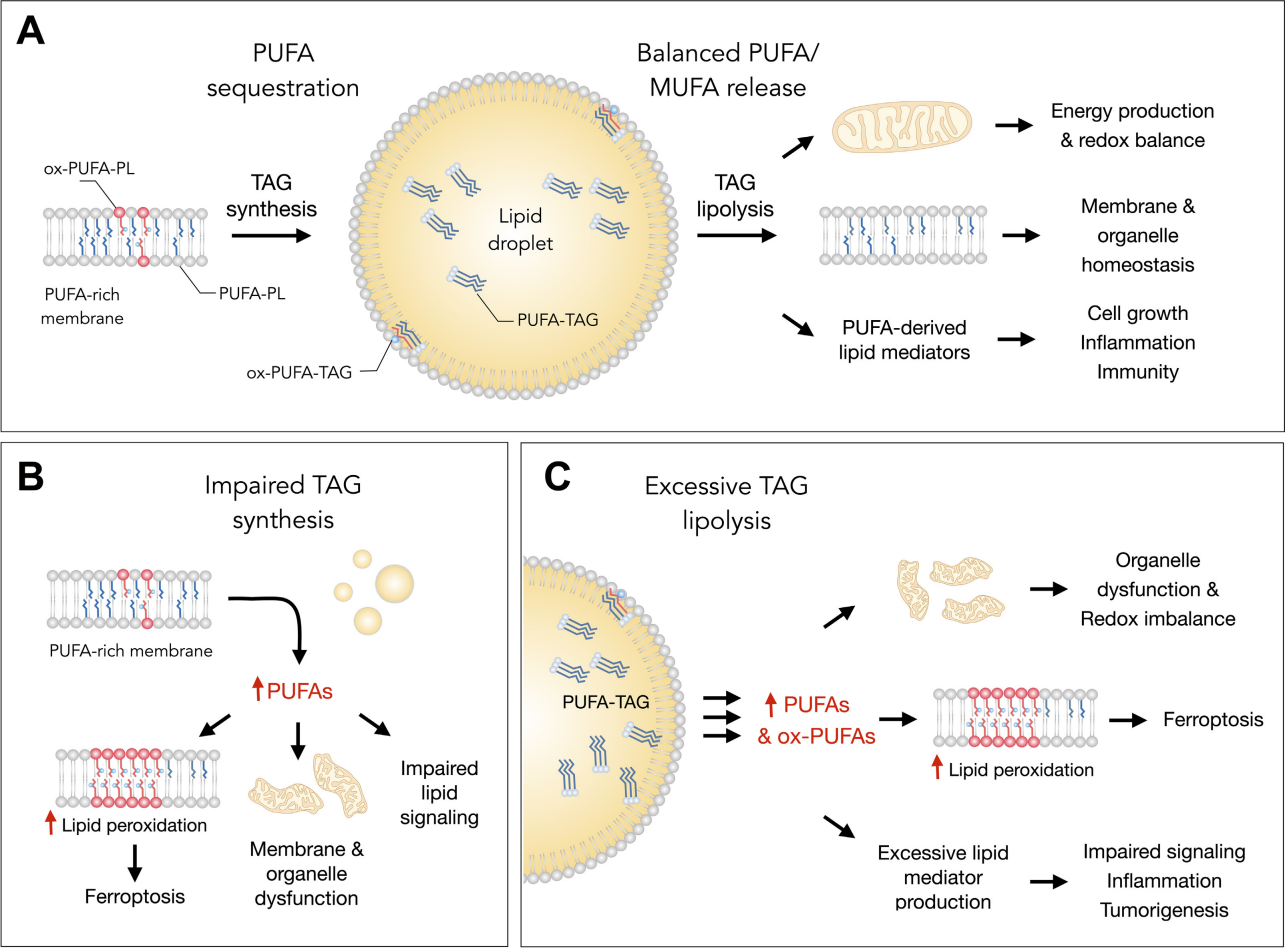
Lipid droplets (LDs) control essential cellular processes and protect from ferroptosis by balancing polyunsaturated fatty acid (PUFA) sequestration and release. **(A)** Under homeostatic conditions, the biosynthesis of triacylglycerols (TAGs) enriched with PUFAs (PUFA-TAGs) and their packaging in LDs may lower the abundance of oxidizable PUFAs esterified in membrane phospholipids (PUFA-PLs). In principle, already oxidized PUFAs esterified in phospholipids (ox-PUFA-PLs) could also be transferred into LDs and stored as oxidized TAGs (ox-PUFA-TAGs). On the other hand, the balanced release of monounsaturated fatty acids (MUFAs) and PUFAs *via* TAG lipolysis maintains proper membrane composition, thereby preventing lipid peroxidation and reducing ferroptosis sensitivity. PUFA release from LDs also supports energy production, mitochondrial function and the redox balance. In addition, lipolysis also delivers PUFAs into oxygenation pathways that convert these FAs into signaling mediators, such as prostaglandins and leukotrienes, which regulate various cellular functions, including cell growth, inflammation and immunity. **(B)** Under various stress conditions, impaired TAG synthesis may lead to an enrichment of membranes with PUFAs that can result in elevated lipid peroxidation and ferroptosis. Excess PUFAs may also overload mitochondria and other organelles, as well as disrupt the biosynthesis of lipid mediators. **(C)** Excessive breakdown of LDs by neutral lipases (at the lipid droplet surface) or acid lipases (in the lysosome) during acute or chronic stress may disrupt organelle function, impair redox signaling and metabolism, and enrich membranes with PUFAs. This can increase lipid peroxidation levels and sensitivity to ferroptosis. The release of excess PUFAs from LDs may also stimulate the production of pro-inflammatory and pro-tumorigenic lipid mediators, which can overstimulate mitogenic, inflammatory and other signaling pathways.

LDs have been implicated in the regulation of FA metabolism and trafficking in various pathophysiological contexts (Bailey et al., 2015; Jarc et al., 2018; Olzmann and Carvalho, 2019; Jarc and Petan, 2020) The importance of LD turnover has been recognized in stressed cells (Henne et al., 2018; Petan et al., 2018) and associated with the protection from the harmful effects of dysregulated lipid and oxidative metabolism. LDs accumulate in cells experiencing various nutrient, energy and redox imbalances caused by hypoxia (Bailey et al., 2015; Schlaepfer et al., 2015), oxidative stress (Liu et al., 2015; Liu et al., 2017; Ioannou et al., 2019), nutrient deficiency (Cabodevilla et al., 2013; Pucer et al., 2013; Rambold et al., 2015; Hariri et al., 2018), exogenous (PU)FA overload (Listenberger et al., 2003; Jarc et al., 2018), acidity (Dierge et al., 2021), inflammation and infection (Brok et al., 2018; Libbing et al., 2019; Jarc and Petan, 2020; Bosch and Pol, 2022). These findings suggest that cells increase LD abundance as a general protective mechanism against stress. Accordingly, a number of studies have reported that blocking LD biogenesis in stressed cells results in cell dysfunction and death (Bailey et al., 2015; Nguyen et al., 2017; Jarc et al., 2018; Dierge et al., 2021; Wilcock et al., 2022). In some conditions, LDs act as “on-demand” reservoirs that fine-tune FA storage and release to control mitochondrial FA oxidation and prevent the build-up of toxic lipids (Liu et al., 2015; Liu et al., 2017; Nguyen et al., 2017). In other conditions, LDs act as antioxidant organelles that sequester membrane PUFAs into their protective neutral lipid core to prevent their oxidation (Bailey et al., 2015). Accordingly, recent evidence suggests that LDs modulate ferroptosis sensitivity by regulating membrane unsaturation through the control of PUFA/MUFA trafficking between the TAG and phospholipid lipid pools (Bai et al., 2018; Brunet et al., 2021; Dierge et al., 2021) (Figure 1). These emerging mechanisms of LD-mediated regulation of mitochondrial function, lipid (per)oxidation and ferroptotic cell death are discussed in the following sections.

## Lipid droplets control lipid fluxes to preserve redox homeostasis and organelle integrity

Recent studies have revealed that LD-mediated control of lipid trafficking is essential for the preservation of redox homeostasis and organelle integrity in various conditions of stress. During severe nutrient deprivation (in the absence of serum and amino acids or glutamine) cells upregulate LD biogenesis, which is driven by FAs derived from bulk autophagy (Rambold et al., 2015; Nguyen et al., 2017). The fact that LDs are formed *de novo* in cells with limited energy sources hints at their essentiality under these conditions. In accordance, diacylglycerol acyltransferase 1 (DGAT1)-mediated LD biogenesis was necessary for the protection of mitochondria against the high flux of autophagy-derived free FAs (Nguyen et al., 2017). Under these conditions, inhibition of DGAT1-mediated TAG synthesis results in the accumulation of toxic levels of acylcarnitines, a transport form of FAs required for mitochondrial uptake, leading to mitochondrial dysfunction. Besides reducing FA lipotoxicity, the sequestration of autophagy-derived FAs in LDs also enables a precise regulation of FA-delivery to mitochondria through the lipolytic activity of adipose triglyceride lipase (ATGL) (Rambold et al., 2015; Nguyen et al., 2017). In conditions of iron deficiency, LDs have been implicated in the regulation of mitophagy, a selective form of autophagy required for the removal of defective mitochondria (Long et al., 2022; Long and McWilliams, 2022). Namely, mitophagy in iron-depleted cells was preceded by massive lipidome remodeling indicative of defects in mitochondrial oxidative and FA metabolism and elevated LD accumulation in the proximity of mitochondria. Inhibition of DGAT1-mediated LD biogenesis led to lysosomal defects and reduced removal of mitochondria *via* mitophagy, suggesting a tight coupling between mitochondrial dysfunction, LD biogenesis and mitophagy (Long et al., 2022). It remains to be investigated how LD-mediated control of lipid homeostasis is connected to the lysosomal system that sustains mitophagy. LDs thus participate in rapid responses to acute stress and are essential for the management of lipid metabolic rearrangements that are required for organelle quality control mechanisms in various contexts.

Cellular and mitochondrial homeostasis are tightly coupled with the continuous control of reactive oxygen species (ROS)-induced stress. In this context, LDs are emerging as pivotal organelles that buffer excessive lipid fluxes that may disrupt the intercellular metabolic coupling between neuronal and glial cells (Teixeira et al., 2020). In general, LDs are rarely found in neurons (Pennetta and Welte, 2018) which have a reduced capacity for mitochondrial FA oxidation (Schönfeld and Reiser, 2013). However, enhanced neuronal activity leads to the accumulation of FAs and to an increase in lipid peroxidation levels, and surprisingly, also to modest LD accumulation, indicating that excess FAs could be partially stored in neuronal LDs (Ioannou et al., 2019). Under these conditions, neuronal mitochondria are highly fragmented and unable to consume excess FAs. Instead, both FAs and lipid peroxides are secreted from neurons as free FAs or as small apolipoprotein E (ApoE)-like particles and subsequently internalized in glial cells, where they accumulate in glial LDs (Ioannou et al., 2019). In accordance, neuronal mitochondrial defects have also been linked with increased production of ROS, which stimulates ApoE-dependent LD accumulation in neighboring glial cells (Liu et al., 2015; 2017). In response to elevated levels of ROS, glial-lactate-driven neuronal lipogenesis is upregulated and excess lipids are transferred *via* ApoE4 to glial LDs, which protects from neurodegeneration (Liu et al., 2017). LD biogenesis is also upregulated in glial cells exposed to glucose deprivation, hypoxia or exogenous FAs (Smolič et al., 2021). LD accumulation in glia is thus stimulated by different stress conditions and regulated by neuronal activity.

Neuronal activity also controls LD breakdown in glia (Ioannou et al., 2019). During excess neuronal activity, increased LD lipolysis and mitochondrial FA oxidation were observed in glia in addition to reduced ROS and lipid peroxidation levels, suggesting that glial cells take-up incoming neuronal-derived FAs and upregulate β-oxidation in order to protect from FA-induced oxidative stress. Therefore, LDs in glial cells provide a dynamic lipid repository that under physiological conditions regulates lipid exchange between neurons and glia through Apo-mediated transport, while under pathological conditions it protects from oxidative stress-induced neurodegeneration (Liu et al., 2015; Liu et al., 2017; Ioannou et al., 2019; Islimye et al., 2022). Intact LD turnover is also essential for proper neural development from neural stem/progenitor cells (NSPCs) (Ramosaj et al., 2021). In adult NSPCs, LDs are highly abundant, but their number is significantly reduced upon NSPC differentiation. However, artificially increasing LD accumulation by the addition of exogenous oleic acid (OA) leads to increased neuronal generation and their better survival. High LD content in NSPCs was associated with increased glycolysis and oxidative phosphorylation, but also with reduced lipid peroxidation and protection against high levels of ROS. In addition, LD lipolysis *via* ATGL was essential for NSPC proliferation. Altogether, LDs play important roles in the nervous system by protecting neurons from acute and chronic damage caused by oxidative stress and membrane lipid peroxidation.

## PUFA sequestration into TAGs reduces lipid (per)oxidation and cell damage

**FIGURE 2:**
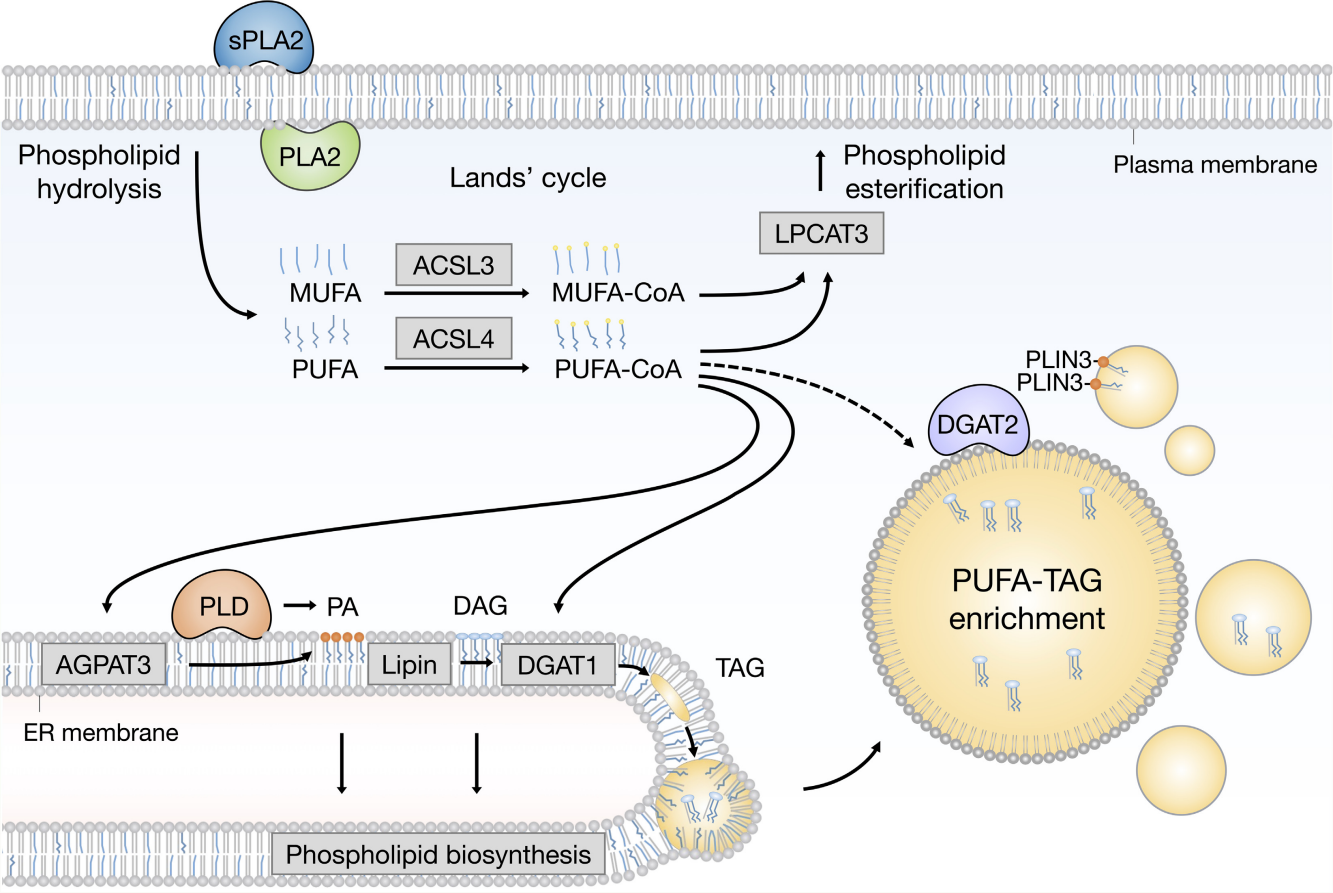
Emerging pathways of PUFA channeling from membranes to lipid droplets (LDs). The redistribution of PUFAs into TAGs stored within LDs can occur *via* at least two conceptually distinct lipid remodeling pathways driven by PLA_2_ and PLD enzymes. PLA_2_-catalysed phospholipid hydrolysis liberates membrane resident PUFAs, which are released into the cytosol. To re-enter any metabolic or remodeling pathways, including the Kennedy pathway of *de novo* phospholipid and TAG biosynthesis, PUFAs have to be first converted into active PUFA-CoA intermediates by CoA synthetase enzymes, such as ACSL4, which shows preference for PUFAs, whereas the activation of MUFAs is preferentially handled by ACSL3. On the other hand, PLD-catalyzed hydrolysis of PUFA-rich phospholipids generates PUFA-containing PA, which remains confined to the 2D space of the membrane bilayer. This in principle enables a more efficient channeling of the PUFA acyl chain into TAGs *via* the Kennedy pathway by the sequential action of lipin and DGAT1 enzymes within the ER membrane. PUFA-PA can also be formed *de novo via* the AGPAT3 enzyme with known preference for PUFAs. In principle, with the help of LD-associated ACSL4, free PUFAs can also be converted into PUFA-TAGs by the DGAT2 enzyme, which can relocate from the ER to LDs to catalyze local LD expansion. In addition, PA formed by PLD can participate in lipid droplet formation and expansion by PLIN3 recruitment. Cofactors and by-products are omitted. ACSL3: acyl-CoA synthetase long chain family member 3; ACSL4: acyl-CoA synthetase long chain family member 4; AGPAT3 (also known as LPAAT3): 1-acyl-*sn*-glycerol-3-phosphate acyltransferase gamma; CoA: coenzyme A; DAG: diacylglycerol; DGAT1: diacylglycerol acyltransferase 1; ER: endoplasmic reticulum; LPCAT3: lysophosphatidylcholine acyltransferase 3; MUFA: monounsaturated fatty acid; PA: phosphatidic acid; PC: phosphatidylcholine; PE: phosphatidylethanolamine; PLA_2_: phospholipase A_2_; PLD: phospholipase D; PLIN3: perilipin 3 PS: phosphatidylserine; PUFA: polyunsaturated fatty acid; sPLA_2_: secreted phospholipase A_2_; TAG: triacylglycerol.

PUFA-containing phospholipids (PUFA-PLs) are prone to oxidation under conditions of oxidative stress. Recent studies suggest that stressed cells activate a protective membrane remodeling mechanism that redistributes PUFAs from the PUFA-PL membrane pool to the PUFA-containing TAG (PUFA-TAG) pool in LDs in order to prevent PUFA oxidation (Bailey et al., 2015; Jarc et al., 2018; Dierge et al., 2021) (Figure 2).

LD biogenesis is strongly induced in central nervous system (CNS) cells of *Drosophila* larvae fed with linoleate rather than with stearic acid, suggesting a stronger LD-mediated buffering of exogenous PUFAs relative to their saturated analogues (Bailey et al., 2015). When the larvae were fed a PUFA-rich diet and then challenged with hypoxia, which induces oxidative stress and increases LD content *per se*, there was a decrease of linoleate-containing phosphatidylcholines (PCs) and phosphatidylethanolamines (PEs), accompanied by a corresponding increase in the linoleate TAG pool (Bailey et al., 2015). This transfer of PUFA acyl chains from membrane phospholipids to TAGs was dependent on the conversion of PC into phosphatidic acid (PA) by phospholipase D (PLD), followed by lipin-mediated dephosphorylation of PA into diacylglycerol (DAG), and its conversion into TAGs by DGAT1 (Figure 2). Knockdown of DGAT1 reduced LD content and suppressed neuroblast proliferation, while increasing PUFA oxidation and accumulation of toxic peroxides *in vitro* and *in vivo* (Bailey et al., 2015). These findings suggest that a precise channeling of PUFAs from membrane phospholipids to drive DGAT1-mediated LD biogenesis is activated in hypoxic neuroblasts to minimize the toxic effects of membrane PUFA oxidation. LDs thus act as antioxidant organelles that sequester membrane PUFAs to reduce their availability for peroxidation.

In line with a protective role of LD biogenesis against oxidative stress and PUFA-induced lipotoxicity, DGAT enzymes have been recently recognized for their roles in coordinating lipid and redox metabolism also in cancer cells (Ackerman et al., 2018; Cheng et al., 2020; Jovičić et al., 2021; Wilcock et al., 2022). In glioblastoma xenografts, targeting DGAT1 disrupted lipid homeostasis, increased acylcarnitine accumulation, and induced oxidative stress, leading to suppressed tumor growth (Cheng et al., 2020). In melanoma cells, DGAT1 inhibition caused excess FA oxidation and ROS generation, accompanied by activation of nuclear factor erythroid 2-related factor 2 (NRF2) signaling, which reduces ROS-mediated cellular damage through upregulation of superoxide dismutase 1 (SOD1) (Wilcock et al., 2022). Combined DGAT1 and SOD1 suppression caused fatal ROS overload and melanoma cell death, as well as suppressed tumor growth. In breast cancer cells, DGAT1-mediated incorporation of PUFAs into TAGs is required to deliver small amounts of PUFAs to oxygenation pathways that convert these FAs into mitogenic lipid mediators (Jovičić et al., 2021). However, when these cells are exposed to high levels of exogenous PUFAs, DGAT1-mediated PUFA sequestration into LDs prevents oxidative stress-dependent cell death (Jarc et al., 2018). DGAT activity is also necessary for maintaining membrane unsaturation levels and preventing clear cell renal cancer cell death and tumor growth during hypoxia (Ackerman et al., 2018). These studies collectively demonstrate that impairing DGAT-mediated LD biogenesis in rapidly proliferating cancer cells disrupts (PU)FA fluxes, which in turn overload mitochondria, alter membrane composition, impair lipid mediator production and cause lethal imbalances in redox defense mechanisms.

Notably, LDs and DGAT enzymes have recently been implicated in the regulation of membrane lipid peroxidation and ferroptosis sensitivity of cancer cells (Dierge et al., 2021). LDs were found to be essential for the suppression of ferroptosis in cancer cells under acidic conditions. LD biogenesis was induced by acidosis and LD accumulation was further augmented by PUFA supplementation. Under these conditions, lipidomic analyses showed that exogenously added PUFAs were preferentially esterified in the TAG pool rather than the phospholipid pool. Longer exposure to exogenous PUFAs and increasing their concentration led to elevated lipid peroxidation and resulted in ferroptotic cell death, possibly due to exceeding the capacity of TAGs to buffer PUFA overload. Importantly, cells treated with DGAT inhibitors lost the ability to accumulate LDs under acidic conditions and PUFA distribution turned from TAGs towards free FAs and membrane phospholipids. Furthermore, DGAT inhibition led to increased cell death, which was reversed by the potent lipophilic radical-trapping antioxidant and ferroptosis inhibitor ferrostatin-1 (Miotto et al., 2020). Finally, in a human tumor xenograft model, a PUFA-rich diet delayed tumor growth in a ferroptosis-dependent manner, and combining a PUFA-rich diet with DGAT1 inhibitor treatment further suppressed tumor progression (Dierge et al., 2021). LD biogenesis is thus important for the prevention of membrane lipid peroxidation and ferroptosis in acidic cancer cells.

LDs may also contain oxidized lipids (Veglia et al., 2017; Zhang et al., 2019; Aldrovandi et al., 2021; Lange et al., 2021), which could sensitize cells to ferroptosis. For example, treating cancer cells with conjugated linoleates, such as α-eleostearic acid (αESA), was found to increase ferroptotic cell death in an LD-dependent manner (Beatty et al., 2021). Mechanistically, the LD-resident long-chain-fatty-acid-CoA ligase 1 (ACSL1) was found to promote αESA incorporation into cellular lipids, including TAGs, which triggered ferroptosis. Lipidomic analyses revealed the presence of oxidized adducts of αESA in the TAG pool. This suggests that LDs may take-up already oxidized PUFAs or serve as sites of lipid (per)oxidation. In accordance with the latter, Lange et al. (2021) have found using an artificial LD model that LD oxidation sensitivity is defined by the unsaturation levels of the PL monolayer and the TAG core, as well as by the ratio of different lipid species, all of which play a significant role in propagation of LD lipid peroxidation. Overall, these findings suggest that PUFA-TAG storage might be a double-edged sword in regulating ferroptosis sensitivity, as it can protect from membrane PUFA oxidation but also act as a source of lipid (per)oxidation, which could propagate to membrane lipids (Beatty et al., 2021). Further research is needed to fully understand the mechanisms involved in TAG oxidation and the role of oxidized TAGs in driving ferroptosis.

## Monounsaturated fatty acids, lipid peroxidation and ferroptosis

**FIGURE 3:**
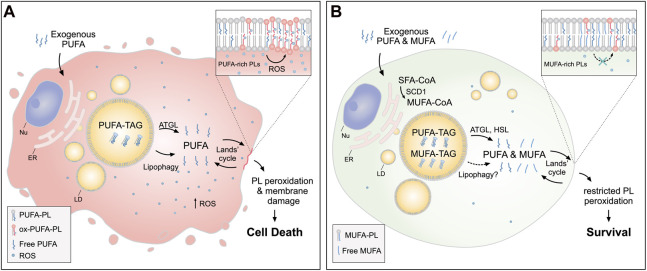
Lipid droplets (LDs) release MUFAs to protect cells from lipid peroxidation and cell death. **(A)** In cells loaded with exogenous PUFAs, the biogenesis of LDs enriched with PUFA-TAGs is increased. Under these conditions, lipolysis *via* ATGL and lipophagy mediate the transfer of PUFAs from LDs to membrane phospholipids (PUFA-PLs), thereby increasing lipid peroxidation and oxidative stress, which can lead to cell death. **(B)** When MUFAs are added simultaneously with PUFAs or synthesized by the cell *via* SCD1, LD are enriched in both species. In this case, lipolysis *via* ATGL and HSL (and likely also lipophagy) liberate both MUFAs and PUFAs from LDs, feeding a more balanced FA mixture for phospholipid acyl chain remodeling pathways (the Lands’ cycle), leading to a reduced abundance of oxidizable PUFAs in membranes and restricted lipid peroxidation. Cofactors and by-products are omitted. ATGL: adipose triglyceride lipase; ER: endoplasmic reticulum; HSL: hormone sensitive lipase; MUFA: monounsaturated fatty acid; Nu: nucleus; Ox: oxidized; PL: phospholipid; PUFA: polyunsaturated fatty acid; ROS: reactive oxygen species; SCD1: stearoyl-CoA desaturase 1; SFA: saturated fatty acid; TAG: triacylglycerol.

Another mechanism employed by cells to protect from lipid (per)oxidation involves the incorporation of less harmful MUFAs in cell membranes in order to reduce PUFA content and membrane unsaturation (Figure 3). The beneficial effect of the monounsaturated OA in the protection from lipotoxicity has been observed in many studies (Listenberger et al., 2003; Ackerman et al., 2018; Chen et al., 2018; Jarc et al., 2018; Lisec et al., 2019; Pérez-Martí et al., 2022) and more recently also linked with ferroptosis resistance (Magtanong et al., 2019; Tesfay et al., 2019; Ubellacker et al., 2020; Brunet et al., 2021; Xie et al., 2022).

Dietary OA reduces renal damage in high-fat diet-fed mice with diabetes by reducing the ER stress caused by saturated FAs (Pérez-Martí et al., 2022). Exogenous OA also alleviates saturated FA toxicity by inducing LD biogenesis and facilitating the incorporation of saturated FAs into TAGs (Listenberger et al., 2003). Nutrient stress together with hypoxia could lead to a harmful increase in FA saturation through inhibition of the oxygen-dependent stearoyl-CoA desaturase 1 (SCD1) (Ackerman et al., 2018). Under hypoxia and serum depletion, TAGs and hormone sensitive lipase (HSL)-mediated lipolysis are an essential source of MUFAs that counters toxic lipid accumulation of ceramides and acylcarnitines through buffering saturated FAs (Ackerman et al., 2018). In breast cancer cells overloaded with PUFAs, which leads to massive accumulation of PUFA-rich LDs driving ATGL-dependent cell death, co-supplementation with OA or membrane remodeling by the group X secreted phospholipase A_2_ (sPLA_2_-X) protects from PUFA lipotoxicity (Jarc et al., 2018). sPLA_2_-X-mediated hydrolysis of lipoproteins and the plasma membrane redirects OA into TAGs, thereby lowering the ratio of PUFA/MUFA released from LDs upon lipolysis by ATGL and reducing cell death (Jarc et al., 2018). Exogenous OA and sPLA_2_-mediated membrane remodeling could therefore act at two levels in parallel to protect from PUFA overload: by direct incorporation of OA into membrane phospholipids and by stimulating a cycle of TAG synthesis and lipolysis that reduces membrane PUFA content (Jarc et al., 2018). In accordance, blocking DGAT-mediated LD biogenesis prevents sPLA_2_-mediated suppression of cell death (Jarc et al., 2018) and may also diminish the protective effect of OA on ferroptotic cell death (Magtanong et al., 2019). Further study is needed to fully understand the mechanistic details and the contributions of these pathways to protection against PUFA toxicity.

Recent studies have shown that the control of MUFA activation and trafficking regulates ferroptotic sensitivity (Magtanong et al., 2019; Ubellacker et al., 2020; Xie et al., 2022). The incorporation of exogenous MUFAs into cellular phospholipids by long chain acyl-CoA synthetase 3 (ACSL3) has been shown to be necessary for the acquisition of a ferroptosis-resistant state in cancer cells, and this process was independent of LD formation (Magtanong et al., 2019). Intercellular MUFA trafficking between adipocytes and cancer cells also contributes to ferroptosis resistance and is mediated by ACSL3 (Xie et al., 2022). In addition, the inhibition of the rate-limiting step of *de novo* MUFA synthesis, which is mediated by SCD1, has been shown to trigger both apoptosis and ferroptosis by decreasing membrane unsaturation, accompanied by ceramide accumulation, and lowering the levels of the endogenous membrane antioxidant coenzyme Q10 (CoQ10), respectively (Tesfay et al., 2019). Targeting MUFA metabolic pathways therefore represents another strategy for inducing ferroptosis in cancer treatment.

Despite evidence that LDs are involved in MUFA trafficking, their role in MUFA-mediated protection from ferroptosis remains unclear (Magtanong et al., 2019; Liang et al., 2022). A recent study in *C. elegans* provides important insights into the potential role of LDs in MUFA-regulated suppression of ferroptosis (Brunet et al., 2021). The study found that LDs are critical for MUFA-induced lifespan extension in *C. elegans* by forming close contact sites with peroxisomes. These “organelle hubs” were required for the maintenance of ether lipid homeostasis, which regulates longevity in part by modulating ferroptosis. The accumulation of dietary MUFAs in ether lipids increases the MUFA to PUFA ratio, reducing the negative impact of PUFA-containing ether lipids and inhibiting ferroptotic cell death. These findings support the idea that LDs are important in the protection from ferroptosis mediated by MUFAs.

LDs are thus involved in several mechanisms that regulate oxidative stress, which are critical for cell survival. They protect cell membranes from lipid oxidation by acting as intracellular buffers that sequester exogenous or membrane-derived PUFAs, and by releasing less harmful MUFAs that contribute to membrane lipid remodeling and homeostasis.

## Lipid droplet breakdown controls PUFA lipotoxicity and ferroptosis sensitivity

It is becoming clear that LDs can regulate the bioavailability of PUFAs and MUFAs, functioning as metabolic hubs that release FAs from neutral lipids “on-demand” to maintain the FA saturation ratio, balance membrane composition, and reduce lipotoxicity (Renne and Hariri, 2021; Bosch and Pol, 2022). However, LD breakdown can be detrimental to cell health under certain conditions, either through the accelerated release of PUFAs from TAGs leading to lipotoxicity or through the activation of pathways involved in lipid metabolism causing metabolic stress. LD breakdown through lipolysis or lipophagy can induce oxidative stress and cell death in conditions of high PUFA burden or hypoxia (Herms et al., 2013; Zechner et al., 2017; Petan et al., 2018; Castelli et al., 2021; Grabner et al., 2021; Schott et al., 2022). Excessive lipolysis has also been associated with various states of lipid overload and cell damage, increasing the pool of free FAs and altering signaling pathways that regulate oxidative metabolism and membrane homeostasis (Haemmerle and Lass, 2018; Li et al., 2022). In contrast, lipophagy has only recently received attention due to its relatively late discovery in 2009 (Singh et al., 2009). However, impaired lipophagy also leads to dysregulated lipid homeostasis through increased lipid accumulation and storage, disrupting the maintenance of energy supply, membrane homeostasis, and mitochondrial function, potentially resulting in cell death (Zhang et al., 2022). The relationship between LD breakdown pathways and lipid peroxidation is currently not well understood, but its role in regulating ferroptosis sensitivity has recently been proposed (Bai et al., 2018; Zou et al., 2019; Tousignant et al., 2020; You et al., 2021).

ATGL-mediated lipolysis in non-adipose tissues has been shown to be involved in the regulation of mitochondrial oxidation, ER stress, turnover of toxic FAs, membrane homeostasis, oxidative stress and FA-mediated signaling. ATGL plays an important role in cellular PUFA distribution and lipotoxicity, which has been linked with cancer cell survival and neurodegeneration. Treatment of breast cancer cells with exogenous DHA resulted in the accumulation of DHA in TAGs and PLs and oxidative stress-induced cell death (Jarc et al., 2018). Blocking ATGL-mediated lipolysis led to a significant LD accumulation with PUFA retention in TAGs, which protected from oxidative stress and cell death, suggesting that lipolysis is responsible for lethal levels of DHA release from LD stores. While the role of ATGL in cancer is still controversial (Petan et al., 2018), ATGL-mediated lipolysis appears to be beneficial in neurons and renal cells (Yang et al., 2020; Lubojemska et al., 2021). In *C. elegans*, ATGL was found to autonomously regulate neuronal LD dynamics and PUFA-mediated neural functions and neurodegeneration (Yang et al., 2020). Mutations in *ATGL-1* and *LID-1* genes, homologs of mammalian ATGL and its co-factor comparative gene identification 58 (CGI-58), were identified as being responsible for pathological LD accumulation in neurons. This finding suggests that lipolysis prevents the appearance of visible LDs in neurons under physiological conditions. Mechanistically, ATGL-1-mediated lipolysis in combination with *de novo* lipogenesis was found to be required for PUFA-mediated touch sensation of neurons, while blocking both pathways was reported to protect against PUFA-induced neurodegeneration. The neurodegeneration was fueled by increased ROS generation and altered PUFA partitioning between phospholipid and TAG species, with reduced PUFA-PL levels and increased PUFA-TAGs. Thus, neuronal lipolysis protects against neurodegeneration by reducing TAG levels. In a *Drosophila* model of diabetes with renal failure, LD lipolysis was found to be beneficial for the preservation of renal cell endocytosis (Lubojemska et al., 2021). A high-fat diet (HFD) led to renal dysfunction with nephrocyte LD accumulation and defects in ER, mitochondria and endocytosis caused by increased levels of circulating lipids. Stimulating nephrocyte lipolysis through ATGL overexpression rescued the HFD-induced endocytosis dysfunction and restored the volumes of mitochondria and ER without increasing lipid peroxidation. In contrast, blocking LD biogenesis through DGAT1 knockdown increased lipid peroxidation levels and failed to rescue mitochondria and the ER, indicating a beneficial role of lipolysis over LD biogenesis in the protection of nephrocytes from HFD lipotoxicity.

Intriguingly, the distribution of PUFAs in the human liver appears to be regulated by patatin-like phospholipase domain-containing protein 3 (PNPLA3) (Luukkonen et al., 2019), which is the closest relative of ATGL within the PNPLA family (Grabner et al., 2021). In human carriers with the PNPLA3-I148M mutation, high PUFA content in liver TAGs and PUFA-deficient lipoproteins secreted by the liver were observed, suggesting PUFA-TAG retention (Luukkonen et al., 2019). Furthermore, PNPLA3 knock-out and PNPLA3-I148M knock-in cells displayed PUFA-induced LD accumulation, while treatment with saturated FAs or MUFAs did not change LD levels, indicating that loss of PNPLA3 results preferentially in PUFA sequestration within TAG. In addition, the distribution of alkyne-linoleic acid was significantly reduced in the phosphatidylcholine fraction but elevated in TAGs in these cells. Under lipolytic conditions, PUFA-containing DAGs accumulated in PNPLA3 knock-out and PNPLA3-I148M knock-in cells in parallel with decreased PUFA-PC levels, suggesting that PNPLA3 is required for the transfer of PUFAs from DAGs to phospholipids and is a major regulator of PUFA distribution in the liver. It is currently unknown whether PNPLA3 also contributes to PUFA-TAG degradation through its TAG lipase activity.

Lipophagy was first described in hepatocytes, where blocking autophagy resulted in LD accumulation (Singh et al., 2009). Since then, the role for autophagy in the regulation of LD turnover, including LD biogenesis and breakdown, has been established in various mammalian cells (Petan et al., 2018; Xie et al., 2020; Schepers and Behl, 2021). In hepatocytes, lipophagy can occur through macrolipophagy, in which autophagosomes selectively target LDs for degradation in lysosomes, or through microlipophagy, in which lysosomes directly engulf LDs. The latter was previously thought to be a conserved pathway of lipophagy limited to yeast, but has recently been confirmed in hepatocytes (Schulze et al., 2020). It is not yet clear which pathway of lipophagy is predominant in hepatocytes, and it appears that this may depend on the type of metabolic stress (Goodman, 2021). Notably, new evidence suggests that (macro)lipophagy may contribute to ferroptosis in hepatocytes (Bai et al., 2018). First, GPX4 inhibition by RAS-selective lethal 3 (RSL3) was found to lead to LD accumulation during the first hours of RSL3 treatment preceding the onset of ferroptotic cell death. This suggests that LD-mediated protective responses may be triggered by the first signals of elevated lipid peroxidation, at a stage, when cell death may still be reversible under physiological conditions. It is currently unknown whether the RSL3-induced LD accumulation is caused by an increase in LD biogenesis or a decrease in LD breakdown. Second, increased lipid storage in hepatocytes has been shown to reduce the level of lipid peroxidation during ferroptosis. Third, silencing the autophagy proteins autophagy-related protein 5 (ATG5) and Ras-related protein 7 (Rab7) has been shown to protect from RSL3-induced ferroptosis, likely by blocking lipophagy and reducing lipid peroxidation levels. Finally, inhibition of lipophagy has also been observed to regulate ferroptosis *in vivo.* Collectively, these findings suggest that lipophagy in hepatocytes is closely connected to ferroptotic cell death.

## Lipid droplets and the sensitivity of therapy-resistant cancer to ferroptosis

Chemical induction of ferroptosis activates major lipid metabolism pathways, including those involved in PUFA-phospholipid membrane remodeling and LD turnover, which may contribute to lipid peroxidation and cell death. For example, induction of ferroptosis by imidazole ketone erastin (IKE) has been shown to alter the expression of enzymes involved in membrane lipid remodeling, LD turnover and lipid signaling in diffuse large B cell lymphoma cancer cells (Zhang et al., 2019). Untargeted lipidomic analyses revealed that IKE-treated cancer cells had significantly lower levels of PUFA-PLs and PUFA-TAGs, while co-treatment with antioxidants or ferrostatin-1 reversed these effects and increased TAG levels. The decrease in PUFA-PLs and PUFA-TAGs upon IKE treatment and upregulation of enzymes involved in phospholipid and TAG remodeling, including group IIF secreted phospholipase A2 (sPLA_2_-IIF), ATGL and lysophosphatidylcholine acyltransferase 4 (LPCAT4), could be associated with activation of protective removal of oxidized PUFA tails from phospholipids and TAGs to prevent oxidative damage. However, the reduction in TAG levels and increase in ATGL expression in IKE-treated cancer cells also suggest activated lipolysis, which could lead to an increased burden of free PUFAs causing cell damage and death. In line with this, inhibition of the ER transmembrane protein inositol-requiring transmembrane kinase/endoribonuclease 1α (IRE1α), a key transducer of the unfolded protein response (Mori, 2009), has been shown to promote PUFA-TAG accumulation and upregulation of genes encoding diacylglycerol acyltransferase 2 (DGAT2) and HSL enzymes, which sensitize cancer cells to starvation (Almanza et al., 2022). This suggests that IREα may be an important modulator of lipolysis-induced PUFA oxidation stress and may contribute to ferroptosis sensitivity.

**FIGURE 4:**
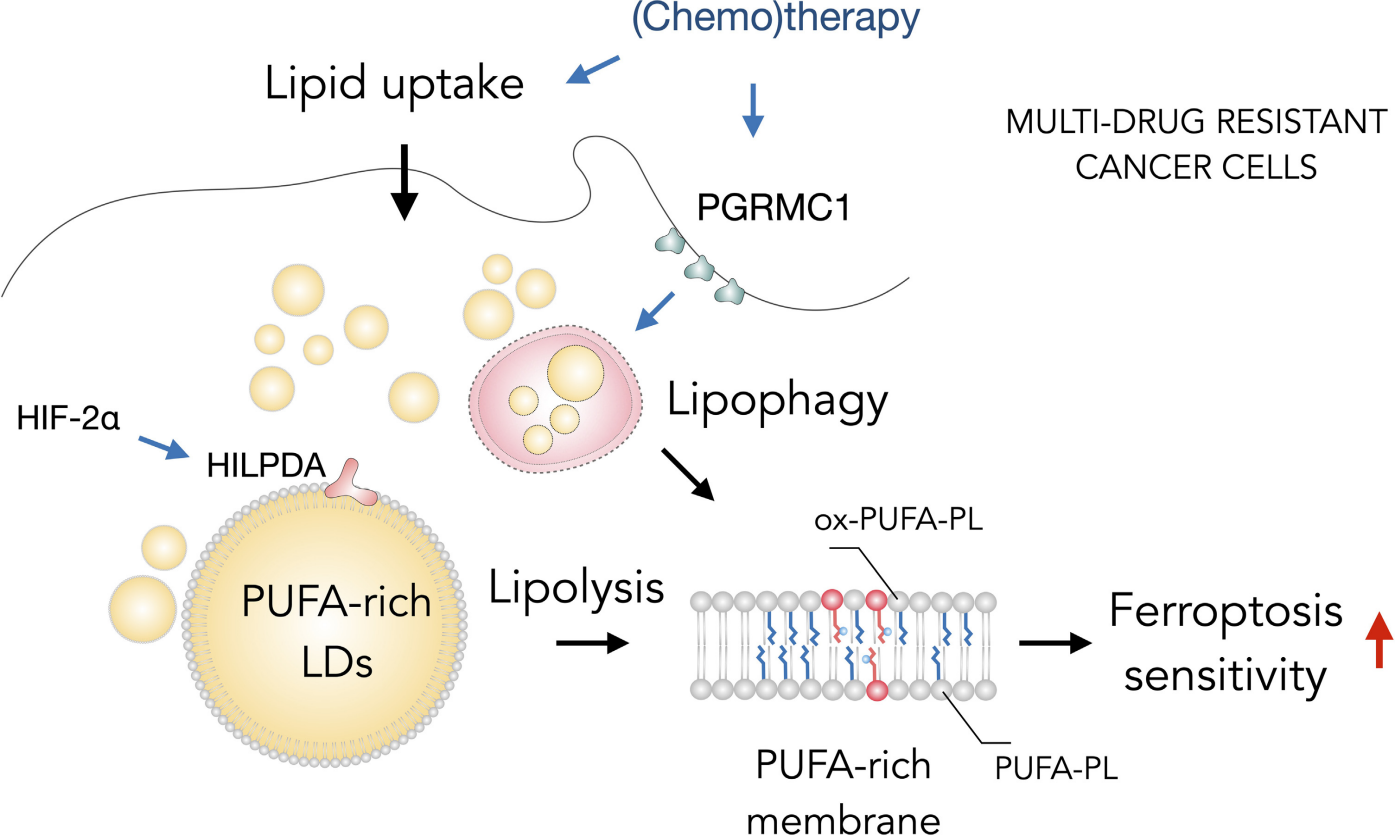
Lipid droplets (LDs) sensitize drug-resistant cancer cells to ferroptosis. The acquisition of multidrug resistant states in cancer cells is accompanied by widespread metabolic remodeling and activation of various signaling pathways, including increased lipid uptake, progesterone receptor membrane component 1 (PGRMC1) overexpression and activation of the hypoxia-inducible factor 2 alpha (HIF-2α)/hypoxia-inducible LD-associated protein (HILPDA) axis, which promote the accumulation of polyunsaturated fatty acid (PUFA)-enriched triglycerides and cholesteryl esters stored within LDs. These LDs may contribute through lipolysis and lipophagy to the enrichment of membrane phospholipids with PUFAs (PUFA-PLs). PUFA-PLs are easily oxidized during oxidative stress giving rise to various oxidized phospholipid species (ox-PUFA-PLs) that impair membrane function and may further propagate membrane lipid peroxidation, which can sensitize cells to ferroptosis.

Interestingly, several types of therapy-resistant cancer cells are particularly sensitive to ferroptosis and LDs can contribute to this sensitivity (Figure 4) (Viswanathan et al., 2017; Angeli et al., 2019; Zou et al., 2019; Petan, 2020; Tousignant et al., 2020; You et al., 2021). Tousignant et al. (2020) recently proposed that ferroptosis hypersensitivity of prostate cancer cells is linked with lipid remodeling caused by androgen-targeted therapy (ATT). ATT-induced changes included a reduction of *de novo* lipogenesis, enhanced lipid uptake, mobilization of storage lipids (TAG and CE) by lipases and phospholipid remodeling with elevated membrane PUFA content and dependence on GPX4 activity (Tousignant et al., 2020). These findings suggest the possibility that LD breakdown is feeding phospholipid synthesis and enrichment with PUFAs and thus mediating ferroptosis sensitivity. Similarly, the highly aggressive clear-cell carcinoma (CCCs) cells were found resistant to a broad range of cancer therapy and highly vulnerable to ferroptosis (Miess et al., 2018). The hypoxia-inducible factor 2α (HIF-2α) was identified as a major regulator of this vulnerability by promoting the selective enrichment of TAG and phospholipid pools with PUFAs through activation of hypoxia-inducible lipid droplet-associated protein (HILPDA) (Zou et al., 2019). HILPDA expression had a profound impact on TAG abundance and enrichment with PUFAs, as well as on PUFA enrichment in PEs, and it also contributed to clear cell morphology. These elevated PUFA levels in LD and membrane lipids are thus associated with high ferroptosis sensitivity. Notably, a role for lipophagy in the regulation of ferroptosis sensitivity of therapy-resistant cancer cells has also been recently described (You et al., 2021). Overexpression of progesterone receptor membrane component 1 (PGRMC1) has been shown to increase ferroptosis sensitivity in paclitaxel-resistant cancer cells through the induction of LD breakdown *via* lipophagy, resulting in increased levels of PUFAs and lipid peroxidation. In summary, targeting ferroptosis together with lipid metabolism in cancer cells may provide a great opportunity in the treatment of aggressive cancers. The close relationship between ferroptosis sensitivity and LD turnover suggests that targeting both pathways could increase the success of cancer therapy.

## Lipid droplets and the production of PUFA-derived signaling mediators

PUFAs are precursors for the production of various signaling lipids, including the AA-derived eicosanoids and the DHA-derived docosanoids. These bioactive lipid mediators, produced by COXs, LOXs and CYP450 monooxygenases, are essential for the modulation of inflammation, the immune response and various homeostatic functions (Dennis and Norris, 2015; Hajeyah et al., 2020; Dyall et al., 2022). Inflammatory cell activation is characterized by LD accumulation and a rapid synthesis and release of numerous lipid mediators that collectively act on target cells to elicit the desired signaling response (Bozza et al., 2011; Dyall et al., 2022). Lipid mediator production is regulated by the availability of particular PUFA species, resident not only in membrane phospholipids, as widely accepted, but also in other lipid pools, including LDs and extracellular lipoproteins (Jarc and Petan, 2020). Recent studies have uncovered that the hydrolysis of neutral lipids stored in LDs by neutral lipases provides PUFAs for lipid mediator production (Dichlberger et al., 2014; Schlager et al., 2015; Gartung et al., 2016; Riederer et al., 2017; Kuo et al., 2018; Jovičić et al., 2021). LDs are thus emerging as important regulators of lipid mediator signaling (Pérez-Chacón et al., 2009; Astudillo et al., 2018; Kita et al., 2018).

Current knowledge on the control of PUFA supply for lipid mediator synthesis is poor and limited to basic biochemical pathways of PUFA distribution between different lipid classes (Pérez-Chacón et al., 2009; Astudillo et al., 2018; Jarc and Petan, 2020). The canonical pathway of stimulus-induced lipid mediator production is driven by membrane phospholipid hydrolysis and release of PUFAs by PLA_2_ enzymes, most notably cPLA_2_α (Dennis and Norris, 2015; Petan, 2020; Murakami et al., 2022). Over the years, several reports have suggested that lipid mediator synthesis may occur at LDs, since cPLA_2_α was found on the LD surface together with COX and LOX enzymes (Dvorak et al., 1993; Bozza et al., 1997; Bozza et al., 2011; Accioly et al., 2008; Wooten et al., 2008; Moreira et al., 2009). However, the evidence for cPLA_2_α action at the LD surface is limited to colocalization studies (D’Avila et al., 2006; D’Avila et al., 2011; Silva et al., 2009). Notably, recent studies suggest that LDs control canonical lipid mediator production by regulating the trafficking of PUFA precursors between neutral and membrane lipid pools (Dichlberger et al., 2014; Jovičić et al., 2021). Accordingly, the TAG lipid pool has emerged as important source of PUFAs for lipid mediator production in different cell types (Bozza et al., 2009; Dichlberger et al., 2014; Schlager et al., 2015; Gartung et al., 2016; Riederer et al., 2017; Kuo et al., 2018; Jovičić et al., 2021; Dierendonck et al., 2022).

Several recent studies have shown that the two major neutral lipases ATGL and HSL participate in lipid mediator production (Dichlberger et al., 2014; Schlager et al., 2015; Gartung et al., 2016; Riederer et al., 2017; Kuo et al., 2018; Sohn et al., 2018; Jovičić et al., 2021; Dierendonck et al., 2022). ATGL-mediated lipolysis has been implicated in eicosanoid synthesis in leukocytes, neutrophils, mastocytes, endothelial and cancer cells (Dichlberger et al., 2014; Schlager et al., 2015; Riederer et al., 2017; Jovičić et al., 2021). Most notably, ATGL deficiency also reduced lipid mediator release in neutrophils *in vivo* (Schlager et al., 2015). In adipocytes, ATGL- and HSL-mediated lipolysis control the production of eicosanoids (Gartung et al., 2016; Sohn et al., 2018) and FA esters of hydroxy FAs (FAHFAs), anti-inflammatory lipid mediators derived from TAG estolides (TAGs with glycerol-bound FAHFAs) (Brejchova et al., 2021). Recent evidence suggests that lipolysis-driven lipid mediator production could be regulated by HILPDA (Dierendonck et al., 2022), an endogenous ATGL suppressor (Grabner et al., 2021). Furthermore, our recent study has shown that DGAT-mediated esterification of PUFAs into TAGs is required for PUFA entry into both the ATGL- and cPLA_2_α-mediated lipid mediator production pathways (Jovičić et al., 2021). In addition, DGAT inhibition completely abolished the canonical PLA_2_-stimulated lipid mediator production, indicating that TAG synthesis is a prerequisite step for lipid mediator synthesis. Finally, lysosomal CE degradation by lysosomal acid lipase (LAL) has been found to drive the synthesis of various lipid mediators in macrophages (Schlager et al., 2017). LAL is also involved in lipophagy (Zechner et al., 2017), however, there is no evidence that LD breakdown by lipophagy is involved in lipid mediator production. Besides LAL, other phospholipases and lipases may also contribute to the process (Jarc and Petan, 2020; Grabner et al., 2021; Murakami et al., 2022; Petan and Manček-Keber, 2022).

Altogether, current evidence suggests that LDs play pivotal roles in lipid mediator signaling. The esterification of PUFAs into TAGs and their subsequent release by different lipases involved in LD breakdown may drive lipid mediator production in various cells and tissues. TAG-derived PUFAs can be either directly oxidized by COX and LOX enzymes or incorporated into membrane phospholipids and then released by PLA_2_s *via* the canonical pathway. LDs are therefore involved in both canonical and non-canonical lipid mediator biosynthesis and may affect various downstream signaling pathways, including inflammation, immunity and tumorigenesis. Further research is needed to confirm the pathophysiological significance of LDs as lipid mediator production hubs.

## Conclusion

LDs play a central role in the trafficking and distribution of intracellular lipids. They are being recognized as regulators of PUFA distribution, as they not only provide membrane building blocks and precursors for lipid mediator production, but also modulate the presence PUFAs in cellular membranes, influencing lipid peroxidation and cell fate. However, our knowledge of how cells regulate LD turnover to fulfill these functions is limited, and the complex coordination between LDs and other cellular mechanisms related to the management of PUFA availability, distribution and oxidation is just beginning to be understood.

Evidence from various species and tissues suggests that LDs protect against oxidative stress and lipid (per)oxidation by taking-up and storing PUFAs in the form of TAGs. However, it is not yet fully understood how the sequestration and release of FAs from LDs is coordinated with changes in redox metabolism aimed at slowing down membrane lipid oxidation and adapting antioxidant surveillance mechanisms to various oxidative and pro-ferroptotic insults. Further research is needed to investigate these processes.

Another important aspect of LD-mediated control of lipid peroxidation is their integration in cellular metabolism. For example, LDs channel FAs to mitochondria and activate signaling pathways in order to sustain respiration and energy production, but also to promote the generation of redox equivalents [e.g., NAD(P)H], which are essential for both redox defense and biosynthetic pathways (Pucer et al., 2013; Rambold et al., 2015; Zechner et al., 2017). In accordance, mitochondrial β-oxidation may also protect cells from ferroptosis (Nassar et al., 2020; Hoy et al., 2021). It will thus be important to investigate the integration of LDs in the wider aspects of cellular metabolism and redox signaling in order to decipher their roles in lipid peroxidation and ferroptosis.

It is also noteworthy that even though the extent of lipid peroxidation within LDs has been poorly studied so far, LDs, including the PUFA-TAG pool, could also be targets for lipid (per)oxidation (Veglia et al., 2017; Zhang et al., 2019; Aldrovandi et al., 2021; Lange et al., 2021). Future research should also examine the mechanisms of PUFA trafficking between the LD phospholipid monolayer and the TAG core, and determine how PUFA oxygenation at the LD surface monolayer vs. the neutral lipid core impacts lipid peroxidation and cell fate.

In summary, it will be interesting to see in future studies how LDs govern (PU)FA trafficking towards various metabolic and signaling fates, including membrane remodeling for organelle homeostasis and protection against harmful lipid (per)oxidation.
