# Erratum: Lipid droplets and polyunsaturated fatty acid trafficking: Balancing life and death

**DOI:** 10.3389/fcell.2023.1175493

**Published:** 2023-03-22

**Authors:** Frontiers Production Office

**Affiliations:** Frontiers Media SA, Lausanne, Switzerland

**Keywords:** lipid droplet, fatty acid, ferroptosis, membrane remodeling, lipid oxidation, lipolysis, phospholipase

Due to a production error, there was a mistake in Figures 1–4 as published. The incorrect low-resolution figures were used. The corrected Figures 1–4 appear below.

**FIGURE 1:**
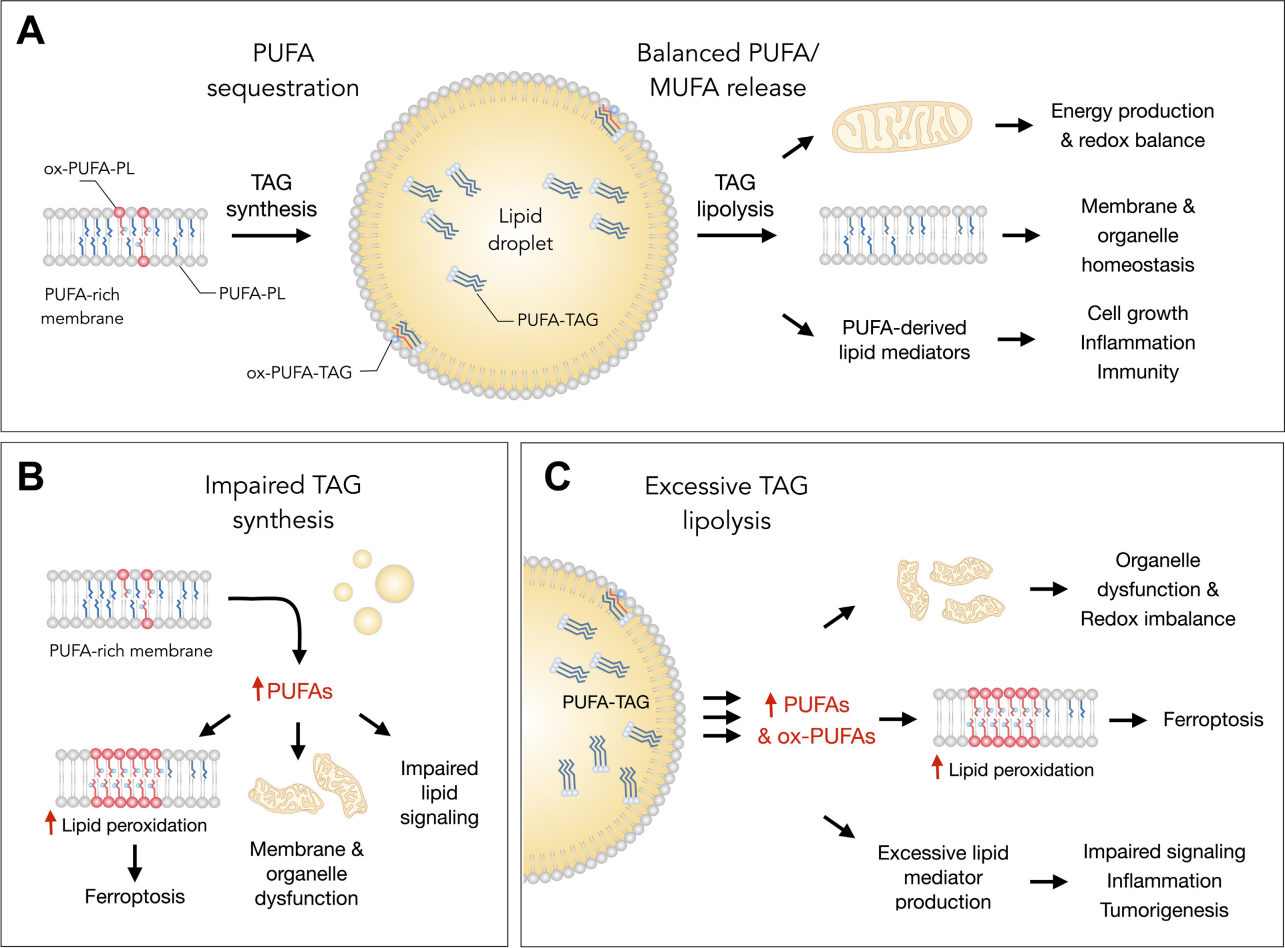
Lipid droplets (LDs) control essential cellular processes and protect from ferroptosis by balancing polyunsaturated fatty acid (PUFA) sequestration and release. **(A)** Under homeostatic conditions, the biosynthesis of triacylglycerols (TAGs) enriched with PUFAs (PUFA-TAGs) and their packaging in LDs may lower the abundance of oxidizable PUFAs esterified in membrane phospholipids (PUFA-PLs). In principle, already oxidized PUFAs esterified in phospholipids (oxPUFA-PLs) could also be transferred into LDs and stored as oxidized TAGs (ox-PUFA-TAGs). On the other hand, the balanced release of monounsaturated fatty acids (MUFAs) and PUFAs *via* TAG lipolysis maintains proper membrane composition, thereby preventing lipid peroxidation and reducing ferroptosis sensitivity. PUFA release from LDs also supports energy production, mitochondrial function and the redox balance. In addition, lipolysis also delivers PUFAs into oxygenation pathways that convert these FAs into signaling mediators, such as prostaglandins and leukotrienes, which regulate various cellular functions, including cell growth, inflammation and immunity. **(B)** Under various stress conditions, impaired TAG synthesis may lead to an enrichment of membranes with PUFAs that can result in elevated lipid peroxidation and ferroptosis. Excess PUFAs may also overload mitochondria and other organelles, as well as disrupt the biosynthesis of lipid mediators. **(C)** Excessive breakdown of LDs by neutral lipases (at the lipid droplet surface) or acid lipases (in the lysosome) during acute or chronic stress may disrupt organelle function, impair redox signaling and metabolism, and enrich membranes with PUFAs. This can increase lipid peroxidation levels and sensitivity to ferroptosis. The release of excess PUFAs from LDs may also stimulate the production of proinflammatory and pro-tumorigenic lipid mediators, which can overstimulate mitogenic, inflammatory and other signaling pathways.

**FIGURE 2:**
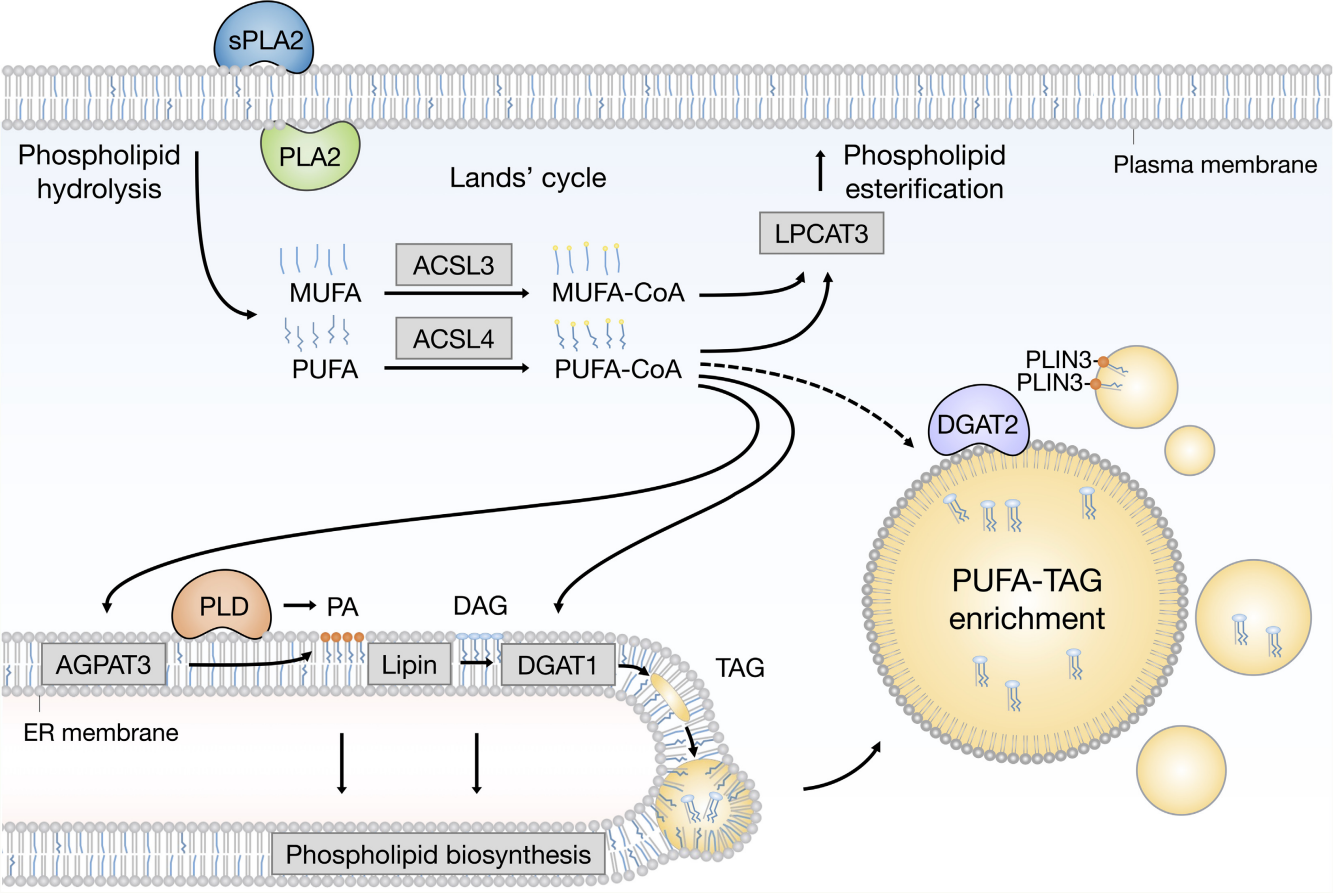
Emerging pathways of PUFA channeling from membranes to lipid droplets (LDs). The redistribution of PUFAs into TAGs stored within LDs can occur *via* at least two conceptually distinct lipid remodeling pathways driven by PLA2 and PLD enzymes. PLA2-catalysed phospholipid hydrolysis liberates membrane resident PUFAs, which are released into the cytosol. To re-enter any metabolic or remodeling pathways, including the Kennedy pathway of de novo phospholipid and TAG biosynthesis, PUFAs have to be first converted into active PUFA-CoA intermediates by CoA synthetase enzymes, such as ACSL4, which shows preference for PUFAs, whereas the activation of MUFAs is preferentially handled by ACSL3. On the other hand, PLD-catalyzed hydrolysis of PUFA-rich phospholipids generates PUFA-containing PA, which remains confined to the 2D space of the membrane bilayer. This in principle enables a more efficient channeling of the PUFA acyl chain into TAGs *via* the Kennedy pathway by the sequential action of lipin and DGAT1 enzymes within the ER membrane. PUFA-PA can also be formed de novo *via* the AGPAT3 enzyme with known preference for PUFAs. In principle, with the help of LD-associated ACSL4, free PUFAs can also be converted into PUFA-TAGs by the DGAT2 enzyme, which can relocate from the ER to LDs to catalyze local LD expansion. In addition, PA formed by PLD can participate in lipid droplet formation and expansion by PLIN3 recruitment. Cofactors and by-products are omitted. ACSL3, acyl-CoA synthetase long chain family member 3; ACSL4, acyl-CoA synthetase long chain family member 4; AGPAT3 (also known as LPAAT3), 1-acyl-sn-glycerol-3- phosphate acyltransferase gamma; CoA, coenzyme A; DAG, diacylglycerol; DGAT1, diacylglycerol acyltransferase 1; ER, endoplasmic reticulum; LPCAT3, lysophosphatidylcholine acyltransferase 3; MUFA, monounsaturated fatty acid; PA, phosphatidic acid; PC, phosphatidylcholine; PE, phosphatidylethanolamine; PLA2, phospholipase A2; PLD, phospholipase D; PLIN3, perilipin 3; PS, phosphatidylserine; PUFA, polyunsaturated fatty acid; sPLA2, secreted phospholipase A2; TAG, triacylglycerol.

**FIGURE 3:**
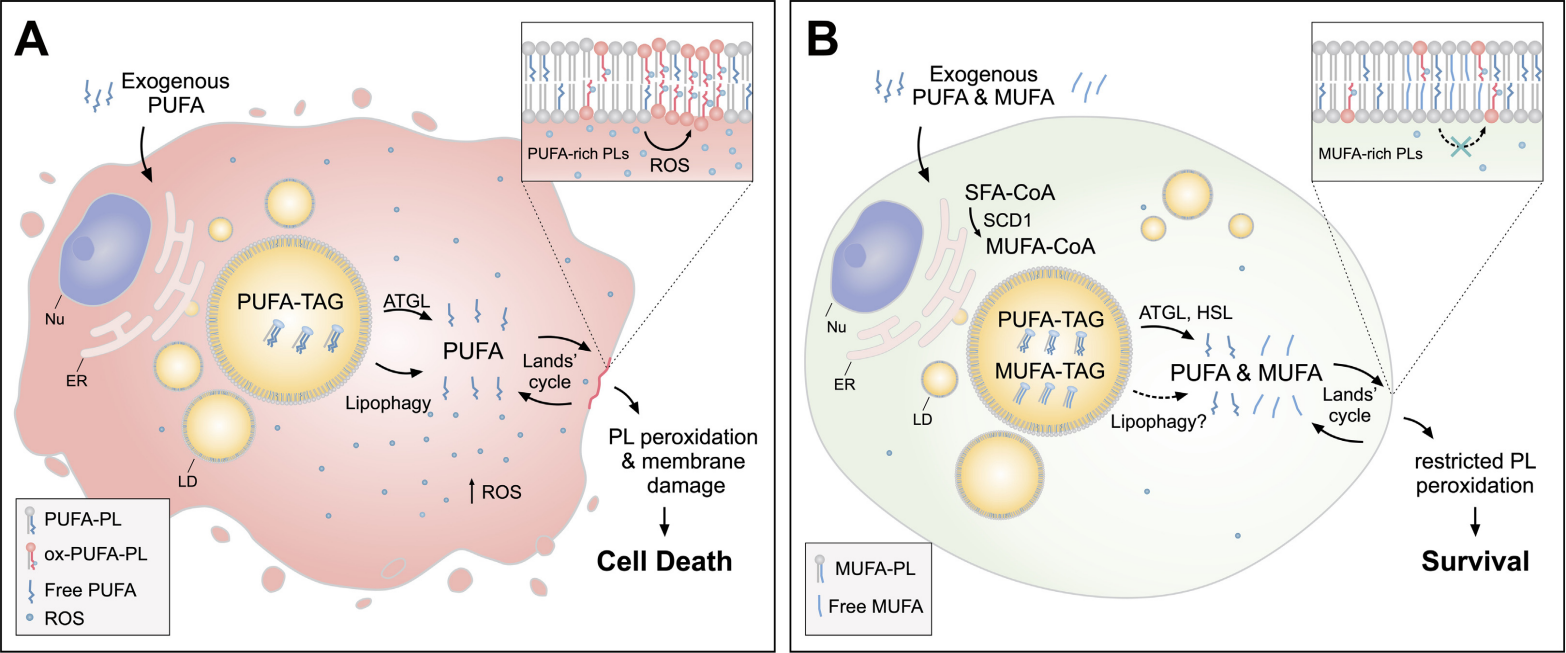
Lipid droplets (LDs) release MUFAs to protect cells from lipid peroxidation and cell death. **(A)** In cells loaded with exogenous PUFAs, the biogenesis of LDs enriched with PUFA-TAGs is increased. Under these conditions, lipolysis *via* ATGL and lipophagy mediate the transfer of PUFAs from LDs to membrane phospholipids (PUFA-PLs), thereby increasing lipid peroxidation and oxidative stress, which can lead to cell death. **(B)** When MUFAs are added simultaneously with PUFAs or synthesized by the cell *via* SCD1, LD are enriched in both species. In this case, lipolysis *via* ATGL and HSL (and likely also lipophagy) liberate both MUFAs and PUFAs from LDs, feeding a more balanced FA mixture for phospholipid acyl chain remodeling pathways (the Lands’ cycle), leading to a reduced abundance of oxidizable PUFAs in membranes and restricted lipid peroxidation. Cofactors and by-products are omitted. ATGL, adipose triglyceride lipase; ER, endoplasmic reticulum; HSL, hormone sensitive lipase; MUFA, monounsaturated fatty acid; Nu, nucleus; Ox, oxidized; PL, phospholipid; PUFA, polyunsaturated fatty acid; ROS, reactive oxygen species; SCD1, stearoyl-CoA desaturase 1; SFA, saturated fatty acid; TAG, triacylglycerol.

**FIGURE 4:**
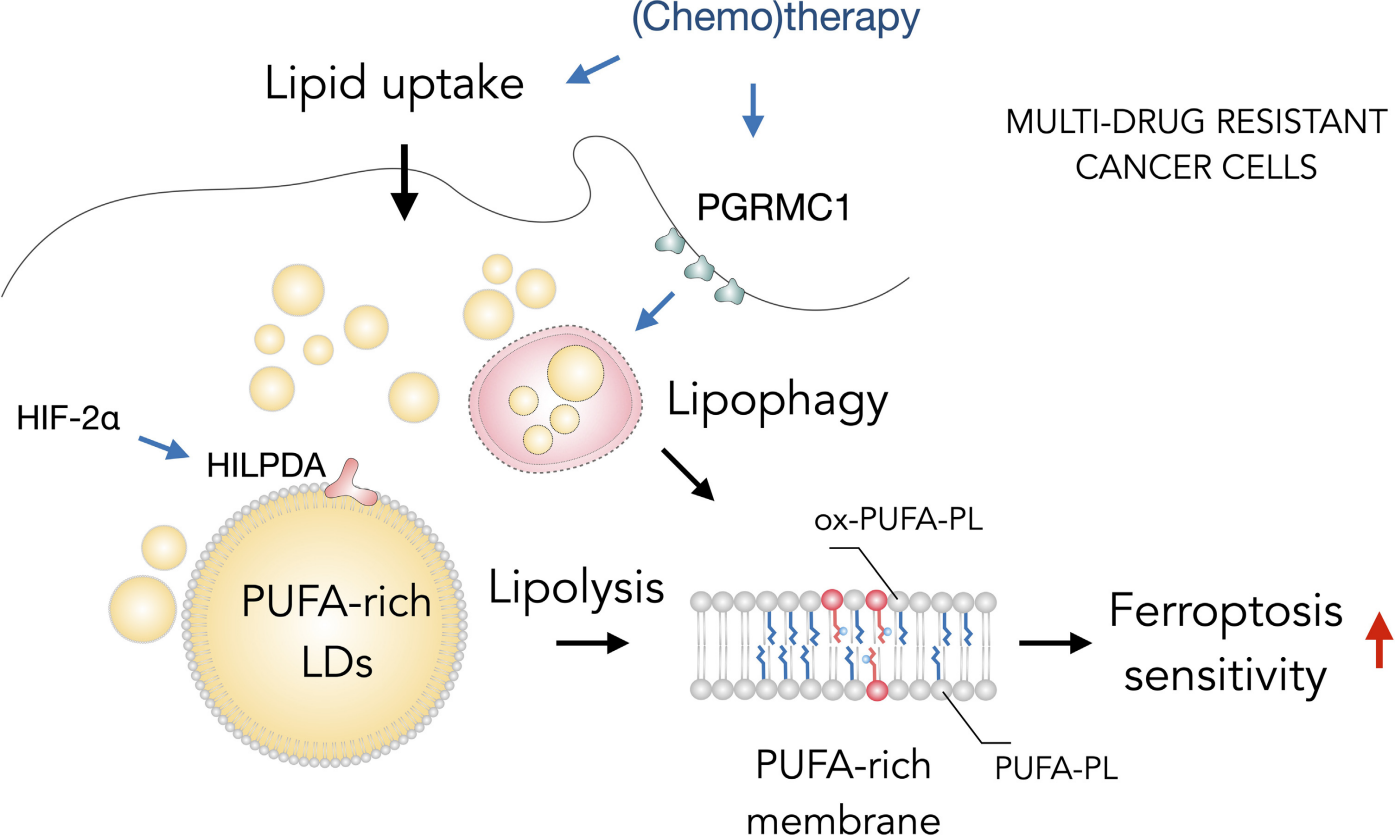
Lipid droplets (LDs) sensitize drug-resistant cancer cells to ferroptosis. The acquisition of multidrug resistant states in cancer cells is accompanied by widespread metabolic remodeling and activation of various signaling pathways, including increased lipid uptake, progesterone receptor membrane component 1 (PGRMC1) overexpression and activation of the hypoxia-inducible factor 2 alpha (HIF-2α)/hypoxia-inducible LD-associated protein (HILPDA) axis, which promote the accumulation of polyunsaturated fatty acid (PUFA)-enriched triglycerides and cholesteryl esters stored within LDs. These LDs may contribute through lipolysis and lipophagy to the enrichment of membrane phospholipids with PUFAs (PUFA-PLs). PUFA-PLs are easily oxidized during oxidative stress giving rise to various oxidized phospholipid species (ox-PUFA-PLs) that impair membrane function and may further propagate membrane lipid peroxidation, which can sensitize cells to ferroptosis.

The publisher apologizes for this mistake. The original version of this article has been updated.

